# Short Term Exercise Induces PGC-1α, Ameliorates Inflammation and Increases Mitochondrial Membrane Proteins but Fails to Increase Respiratory Enzymes in Aging Diabetic Hearts

**DOI:** 10.1371/journal.pone.0070248

**Published:** 2013-08-01

**Authors:** Amy Botta, Ismail Laher, Julianne Beam, Daniella DeCoffe, Kirsty Brown, Swagata Halder, Angela Devlin, Deanna L. Gibson, Sanjoy Ghosh

**Affiliations:** 1 Department of Biology, IK Barber School of Arts and Sciences, University of British Columbia-Okanagan, Kelowna, British Columbia, Canada; 2 Department of Pharmacology and Therapeutics, University of British Columbia, Vancouver, British Columbia, Canada; 3 Department of Pediatrics, University of British Columbia, Vancouver, British Columbia, Canada; Max-Delbrück Center for Molecular Medicine (MDC), Germany

## Abstract

PGC-1α, a transcriptional coactivator, controls inflammation and mitochondrial gene expression in insulin-sensitive tissues following exercise intervention. However, attributing such effects to PGC-1α is counfounded by exercise-induced fluctuations in blood glucose, insulin or bodyweight in diabetic patients. The goal of this study was to investigate the role of PGC-1α on inflammation and mitochondrial protein expressions in aging *db/db* mice hearts, independent of changes in glycemic parameters. In 8-month-old *db/db* mice hearts with diabetes lasting over 22 weeks, short-term, moderate-intensity exercise upregulated PGC-1α without altering body weight or glycemic parameters. Nonetheless, such a regimen lowered both cardiac (macrophage infiltration, iNOS and TNFα) and systemic (circulating chemokines and cytokines) inflammation. Curiously, such an anti-inflammatory effect was also linked to attenuated expression of downstream transcription factors of PGC-1α such as NRF-1 and several respiratory genes. Such mismatch between PGC-1α and its downstream targets was associated with elevated mitochondrial membrane proteins like Tom70 but a concurrent reduction in oxidative phosphorylation protein expressions in exercised *db/db* hearts. As mitochondrial oxidative stress was predominant in these hearts, in support of our *in vivo* data, increasing concentrations of H_2_O_2_ dose-dependently increased PGC-1α expression while inhibiting expression of inflammatory genes and downstream transcription factors in H9c2 cardiomyocytes *in vitro*. We conclude that short-term exercise-induced oxidative stress may be key in attenuating cardiac inflammatory genes and impairing PGC-1α mediated gene transcription of downstream transcription factors in type 2 diabetic hearts at an advanced age.

## Introduction

Type 2 diabetes (T2D) has become common in the elderly. In Canada, currently around 23% of the population above 65 years of age suffer from diabetes, predominantly type 2 diabetes [1] Cardiovascular disease is the leading cause of morbidity and mortality among such patients. Other common features of T2D are persistent, low-grade inflammation and mitochondrial deficiency in multiple organs including the heart and skeletal muscle [2]. A key player involved in both mitochondrial dynamics and inflammation is peroxisome proliferator-activated receptor gamma coactivator 1-alpha (PGC-1α) [3].

PGC-1α is a coactivator that increases the transcriptional activity of multiple pathways which control inflammation, mitochondrial respiration and biogenesis [4]. Expression of more than 70% of all the subunits of the electron transport chain and all of the enzymes of the Krebs cycle are controlled by PGC-1α in the heart [5]. Currently, PGC-1α mediated mitochondrial protein synthesis and anti-inflammatory benefits are reported to be dependent on insulin sensitivity or glycemic improvements in skeletal muscle or adipose tissues [6], [7], [8]. However, like the aforementioned organs, the myocardium is also insulin sensitive and is under the control of PGC-1α during its development and pathology [5]. Although the majority of cardiovascular effects of exercise is dependent on improvements in blood glucose or insulin, exercise-mediated benefits in human diabetes can be independent of such changes in glycemic parameters [9], [10], [11]. Likewise, with moderate exercise, we previously reported antioxidant benefits in the aorta and the heart of young *db/db* mice without having changes in blood glucose or insulin levels [12], [13]. As PGC-1α is believed to signal via insulin [14], [15], whether PGC-1α might be involved in cardiac benefits independent of changes in blood glucose or insulin in diabetes is currently unknown.

The goal of this study was to investigate the relationship between exercise-induced elevation of cardiac PGC-1α with cardiac inflammation and mitochondrial status in aging *db/db* mice hearts independent of alterations in glycemic parameters. We report that elevated cardiac PGC-1α expression following short term, moderate intensity exercise was associated with a reduction in both systemic and cardiac-specific inflammation in aged *db/db* mice without alterations in body weight, blood glucose or insulin. An induction of PGC-1α in these hearts was accompanied by elevated mtDNA but reduced expression of downstream transcriptional activators of PGC-1α. Such a defect could have led to elevated levels of mitochondrial membrane proteins but reduced respiratory enzyme expression in exercised *db/db* hearts. As these chronically diabetic hearts also show augmented oxidative damage and low cardiac antioxidants, we further demonstrate that elevated oxidative stress could increase PGC-1α itself but lower PGC-1α mediated expression of pro-inflammatory cytokines and downstream transcription factors in cardiomyocytes *in vitro*. We conclude that short-term exercise induced oxidative stress might be key in attenuating cardiac inflammatory genes but could also impair PGC-1α mediated gene transcription of downstream transcription factors post-exercise in type 2 diabetic hearts at an advanced age.

## Materials and Methods

### 2.1 Animal Models and Experimental Protocols

Male *db/db* mice become obese by 1 month, develop diabetes by 2 months and die within 10 months of age (http://jaxmice.jax.org/strain/000642.html). Therefore, at 80% of their lifespan, 8-month old *db/db* mice could be considered to represent the late stages of T2D at an advanced age. The following investigation conformed to the an approved animal care protocol by the Animal Care Committee (ACC) of the University of British Columbia. Six-week old male *db/db* and age-matched male wild type (*Wt*, C57BLKS/J) mice were purchased from Jackson Laboratories and maintained under a 12 h light/dark cycle until they were at least 32 weeks of age and the *db/db* mice had been hyperglycemic for at least 22 weeks. Groups of *db/db* and *Wt* mice were randomly placed into sedentary and moderate intensity exercise (Exe) groups. Exe mice were gradually trained to run on a motorized exercise wheel system (Lafayette Instrument Co, USA). Exercise intensity was increased over the first week to a target of 1 h of daily exercise at a speed of 5.2 m/min. Mice were exercised for 5 days/week for the next 2 weeks. Sedentary *db/db* or *Wt* mice were placed in non-rotating wheels for the same duration. Animals were housed in groups of 4 per cage, and had free access to food and water throughout the entire study. At the end of the experimental protocols, the animals were anesthetized with isoflurane followed by sacrifice by CO2 inhalation. Blood was collected and glucose measured with a glucometer (Accuchek). A section of the heart was processed for electron and light microscopy. Rest of the left ventricle and freshly separated plasma were flash-frozen in liquid nitrogen and stored at −80°C.

### 2.2 Total Nitrate/nitrite Assay

Total tissue nitrite/nitrate was measured by a commercial kit (Cayman Chemicals, USA) according to Griess protocol. Protein assays were performed according to the Bradford method (Biorad).

### 2.3 Western Blots

Western blotting was performed as described previously [16]. Flash-frozen sections of the left ventricle were homogenized in an ice-cold homogenization buffer, followed by centrifugation and separation of the supernatant. Proteins were quantified and denatured. Samples (50 ug) were then run on sodium dodecyl sulfate polyacrylamide gel electrophoresis (SDS-PAGE) (10%). After transfer, the nitrocellulose membranes were blocked overnight in 5% skim milk in Tris-buffered saline containing 0.1% (vol/vol) Tween-20 (TBS-T). Following three washes, membranes were incubated for 2 hours at room temperature with the primary antibodies against iNOS, TOM 70, VDAC-1 (Santa Cruz Biotechnology, CA, USA). MitoProfile Total OXPHOS rodent antibody cocktail (MitoSciences, Eugene, OR) was used to identify the relative abundance of respiratory units, particularly the five electron transport chain complexes in all groups [17]. This cocktail has been used previously to ascertain the relative abundance of all the 5 oxidative phosphorylation complexes in the heart, and contains five monoclonal antibodies raised against Complex I subunit NDUFB8 (NADH dehydrogenase [ubiquinone] 1 beta subcomplex subunit 8), Complex II subunit 30 kDa, Complex III core protein 2, Complex IV subunit I and Complex V alpha subunit in mouse. Following three washes in TBS-T, membranes were incubated for 2 hours at room temperature with the appropriate alkaline phosphatase conjugated secondary antibodies against rabbit, goat and mouse IgGs (Santa Cruz Biotechnology, CA, USA). For loading controls, goat anti-human β-actin was used. Multiple antibodies were probed on the same blot using the Restore Plus stripping buffer (Thermo Scientific, USA) for 10 minutes each time according to the manufacturer’s instructions [18]. Detection was performed by using an Amersham ECL kit. The signals were digitized using the Chemigenius System (Syngene, USA). Band density was quantified using Image J (NIH), and was expressed as a ratio to β-actin signal in arbitrary units (A.U.).

### 2.4 mRNA Analysis

mRNA levels of various proteins were quantified using the Real-Time PCR ΔΔCT method using the CFX96 platform (Biorad). Total RNA was purified using Qiagen RNEasy kits (Qiagen) according to the manufacturer’s instructions. cDNA was synthesized with the iScript cDNA Synthesis Kit (Bio-Rad) [19]. Quantitative PCR reactions were performed using Bio-Rad CFX Manager 2.0 and Sso Fast Eva Green Supermix (Bio-Rad). All primers were synthesized by Integrated DNA Technology (IDT), Canada. Primer efficiencies were verified according to the minimum information for publication of quantitative real-time PCR experiments (MIQE) guidelines. The primer sequences for mRNA analysis are given in Table S1 and S2 in File S1. Expression of 18S rRNA was used to normalize gene expression. Quantification of gene expression was carried out using the CFX manager software version 1.6.541.1028 (Bio-Rad).

### 2.5 Immunofluorescence

Immunofluorescence was performed as described previously [16]. Rabbit polyclonal antibodies directed against F4/80 (Cedarlane), SOD2 (Santa Cruz Biotechnology), VDAC-1 and CoxIV (ABMGood, Richmond, CA) were used as primary antibodies with anti-rabbit Dylight594 or 488 (ABMGood) as secondary antibodies. DAPI was used as a nuclear counterstain where indicated. Images were obtained with an Olympus IX81 microscope with Texas Red, FITC and DAPI filters. After acquisition of at least 5 frames per sample (n = 5 per experimental group) by a blinded observer, Image J (NIH) was used to calculate immunopositivity across cardiac sections for each group.

### 2.6 Mitochondrial DNA Density

Mitochondrial density was estimated as previously described [18], [20]. In brief, it was determined by quantifying the mitochondrial encoded cytochrome b gene (*mt-Cytb*) copy number relative to the nuclear encoded beta-actin gene (*Actb*) copy number using real-time PCR. Genomic DNA was extracted from heart samples using the DNeasy Kit (Qiagen). The following primers were used: for *mt-Cytb*, CytbF: 5′-CCACTTCATCTTACCATTTATTATCGC-3′ and CytbR: 5′-TTTTATCTGCATCTGAGTTTAATCCTGT-3′; and for *Actb*, ActbF: 5′-CTGCCTGACGGCCAGG-3′ and ActbR: 5′-GAAAAGAGCCTCAGGGCA T-3′. The following FAM-labelled probes were used: for *mt-Cytb*, cytbFAM: 5′-FAM-AGCAATCGTTCACCTCCTCTTCCTCCAC-3′ and for *Actb*, ActbFAM: 5′- FAM-CATCACTATTGGCAACGAGCGGTTCC-3′. Copy numbers were quantified using TaqMan PCR reagents and an ABI 7500 Real Time PCR System (Applied Biosystems).

### 2.7 Transmission Electron Microscopy of Mitochondria

Ultrastructural evaluation of cardiac mitochondria was carried out using transmission electron microscopy. Left ventricular tissues were fixed in 1.5% glutaraldehyde and paraformaldehyde, and cut into several blocks of approximately 1 mm^3^ size. These blocks were postfixed with 1% osmium tetraoxide, and dehydrated using graded concentrations of ethanol (50–100%). Blocks were then embedded in molds, polymerized, and sectioned at around 100 nm. Sections were stained with 1% uranyl acetate and Reynold's lead citrate. Images of the longitudinal sections were obtained with an Hitachi H7600 electron microscope. The mitochondrial number across the myofibrillar areas was estimated by blind counting of four sections per sample (n = 4 for each experimental group) at 25,000 and 16,000× for determination of mitochondrial number and fission characteristics respectively.

### 2.7 TBARS Assay

Lipid peroxidation in cardiac tissue was estimated by thiobarbituric acid-reactive substances (TBARS) assay as demonstrated previously [21]. Flash-frozen ventricular tissue was homogenized under liquid nitrogen with 1% phosphoric acid and 0.6% thiobarbituric acid (TBA). The mixture was then heated in a boiling water bath for 1 h in the presence of 0.4% butylated hydroxytoluene, an antioxidant to prevent air oxidation of the boiling tissue. After cooling, 1∶2 adduct of malondialdehyde and TBA was extracted in 4 ml of *n*-butanol and its absorbance measured at 540 nm against 1,1,3,3-tetramethoxypropane was used as the standard. Values were expressed as per mg protein.

### 2.8 Analysis of Cytokines and Chemokines

Multiplexing analysis of cytokines, chemokines and growth factors were performed not only by Eve Technologies Corp. (Calgary, AB, CA) using the Luminex™ 100 system (Austin, TX, USA) but also by using the MILLIPLEX Mouse Cytokine/Chemokine kit (Millipore, St. Charles, MO, USA) in accordance with the manufacturer's protocol. The assay sensitivities of these markers ranged from 0.3–63.6 pg/mL. Results were expressed as pg/ml of plasma.

### 2.9 Cell Culture and PGC-1α Knockdown Studies

H9C2, a rat cardiomyoblastic cell line was purchased from American Type Culture Collection (ATCC; CRL-1446). Cells were maintained in DMEM supplemented with 10% fetal bovine serum, 2% penicillin-streptomycin, at 37°C in a humidified atmosphere of 5% CO2. H9C2 cells were differentiated into an adult cardiomyocyte phenotype by reducing the media serum to 1% and by daily addition of 0.1 µM retinoic acid for 4 days [22]. Knockdown of PGC-1α expression was achieved using RNA interference techniques. Briefly, differentiated, serum starved H9C2 cells were treated with RNAi for either PGC-1α (catalog # SC-72151) or control scrambled RNAi (catalog# SC-37007) following manufactures instructions (Santa Cruz Biotechnology, Tx, USA). After 24 hours recovery, a subset of cells were treated with 200 ng/ml lipopolysaccharide (LPS, L2880; Sigma Aldrich) with/without varying concentrations of hydrogen peroxide (H_2_O_2_, H1009; Sigma Aldrich) for 12–24 hours. RNA was extracted using RiboZOL extraction reagent (Amersco, OH, USA) following manufactures instructions for qPCR. The primer sequences specifically used for rat H9c2 cells used are given in Table S2 in File S1.

### 2.10 Statistical Analysis

Results were expressed as mean ± standard error. Two way ANOVA with multiple comparisons using Bonferroni’s tests were used in all tests. The level of statistical significance was set at p<0.05.

## Results

### 3.1 Short-term Exercise Augments PGC-1α Expression in Aged *db/db* Hearts without Changing Body Weight, Blood Glucose or Plasma Insulin

**Figure 1:**
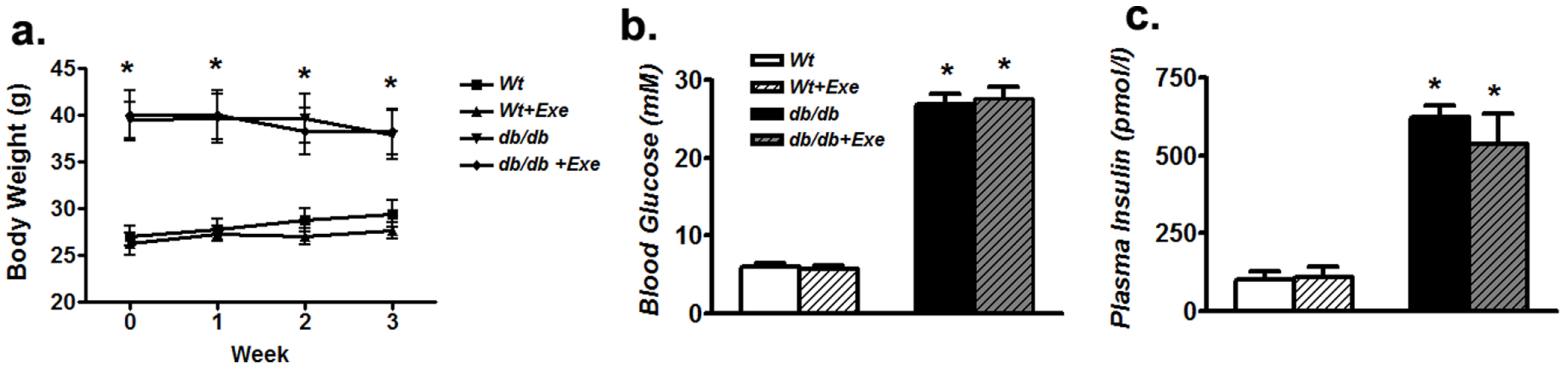
Short-term moderate intensity exercise does not alter body weight or glycemic parameters in aging *db/db* mice. (a) Body weight over three weeks of exercise regimen (b) Fasting blood glucose at the end of the exercise regimen (c) Fasting plasma insulin at the end of the exercise regimen. Data was analyzed using two-way ANOVA with Bonferroni tests, *p*<0.05 (n = 6). *P<0.05 versus corresponding *Wt* group. Abbreviations:pmol, picomolar, Exe, exercise.

**Figure 2:**
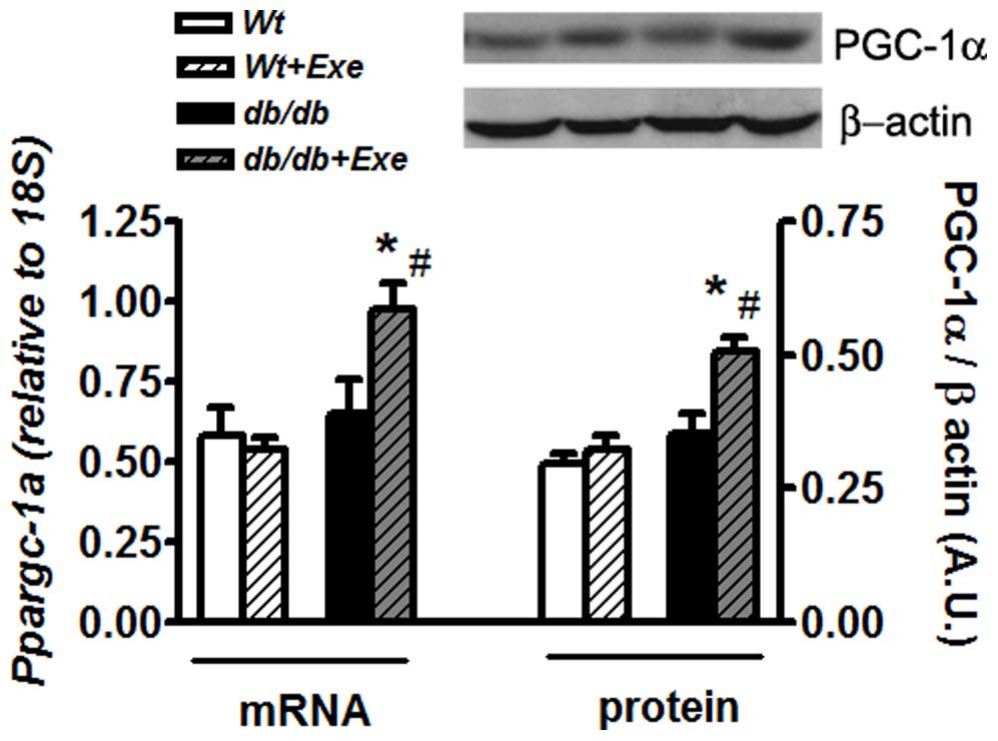
PGC-1α mRNA and protein are upregulated with short term exercise in *db/db* mice hearts. PGC-1α mRNA (left panel) and protein (right panel) levels in Wt and *db/db* mice hearts with or without exercise. Real time PCR analysis of PGC-1α mRNA was normalized to 18S RNA, while PGC-1α protein was normalized against β-actin. Inset: representative bands of PGC-1α and β-actin proteins from the same blot. Data was analyzed using two-way ANOVA with Bonferroni tests, *p*<0.05 (n = 6). *P<0.05 versus corresponding *Wt* group; ^#^P<0.05 versus corresponding sedentary group. Abbreviations:PGC-1α, peroxisome proliferator-activated receptor gamma coactivator 1-alpha, Ppargc-1a, gene nomenclature for PGC-1α; A.U., arbirary units.

Three weeks of exercise did not significantly alter body weight at any time point (Figure 1a). Such a regimen was also ineffective in altering the blood glucose and insulin levels in *db/db* mice but they were still elevated compared to Wt at the end of the study (Figure 1b and c). However, such an exercise regimen was able to increase both mRNA and protein levels of cardiac PGC-1α in *db/db* mice hearts (Figure 2). There was no difference in PGC-1α expression between sedentary Wt and *db/db* hearts (Figure 2).

### 3.2 Induction of PGC-1α is Associated with Reduction in Systemic and Cardiac Inflammation in *db/db* Mice

**Figure 3:**
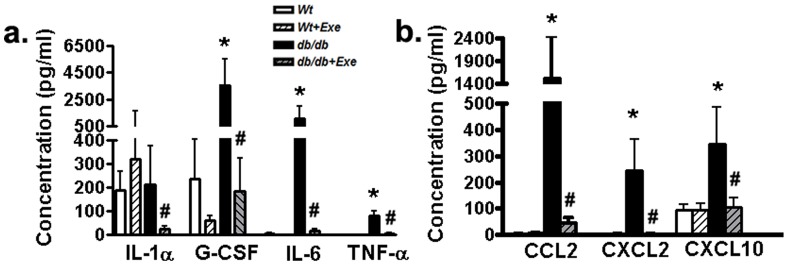
Induction of PGC-1α is associated with reduction in systemic inflammation in *db/db* mice. (a) Circulating proinflammatory cytokines and (b) macrophage/monocyte trafficking chemokines as analyzed in plasma using a multiplex array and quantified in pg/ml. Data was analyzed using two-way ANOVA with Bonferroni tests, *p*<0.05 (n = 6). *P<0.05 versus corresponding *Wt* group; ^#^P<0.05 versus corresponding sedentary group. Abbreviations: IL-1α, Interleukin-1 alpha; G-CSF, Granulocyte-colony stimulating factor; IL-6, Interleukin-6; TNF-α, Tumor necrosis factor alpha; CCL2, Chemokine (C-C motif) ligand 2; CXCL2, Chemokine (C-X-C motif) ligand 2; CXCL10, Chemokine (C-X-C motif) ligand 10.

Overall, long-term T2D was associated with a general increase in proinflammatory cytokines and chemokines in the plasma of aging *db/db* mice compared to Wt mice (Figure 3a and b). A short-term induction of PGC-1α in *db/db* hearts was associated with reduced systemic inflammation as evident from lower plasma proinflammatory cytokine levels [plasma interleukin (IL) 1α, granulocyte colony stimulating factor (G-CSF), IL-6 and TNFα] (Figure 3a). A reduction in proinflammatory cytokines in exercised *db/db* mice was matched by a parallel reduction in the levels of CCL2, CXCL2 and CXCL10, which are chemokines involved in monocyte and macrophage trafficking (Figure 3b).

**Figure 4:**
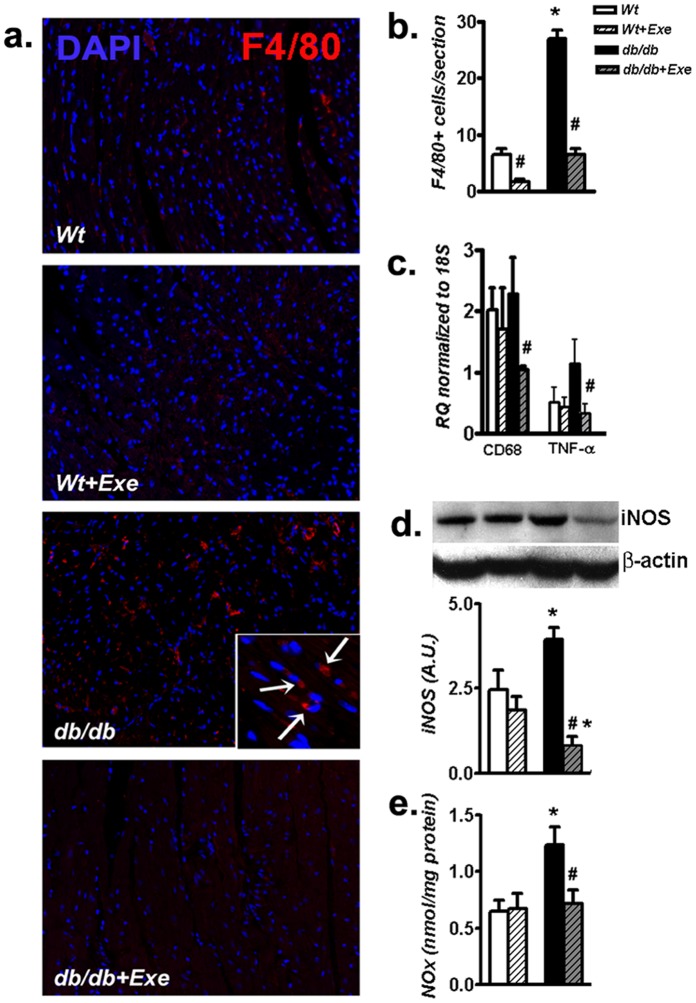
Cardiac inflammation is reduced in *db/db* mice undergoing short-term, moderate intensity exercise. (a) Representative micrographs of heart sections probed with rabbit anti-F4/80 Ab for macrophages, stained with Alexafluor594 labeled rabbit secondary Ab and co-stained with DAPI to visualize nuclei. Magnification 200×. Inset: *db/db* heart sections demonstrating infiltrating macrophages at 630× magnification. (b) Quantification of F4/80^+^ cells in the ventricle. (c) Real time PCR analysis of the expression of cardiac CD68 and TNF-α normalized to 18S RNA. (d) Western blot analysis of cardiac iNOS to β-actin ratio expressed in arbitrary units. Inset: representative bands of respective proteins from the same blot. (e) Total cardiac nitrate/nitrite levels (NOx ) as measured by using a commercial kit. Data was analyzed using two-way ANOVA with Bonferroni tests, *p*<0.05 (n = 6). *P<0.05 versus corresponding *Wt* group; ^#^P<0.05 versus corresponding sedentary group. Abbreviations:TNF-α, Tumor necrosis factor alpha, DAPI, 4′,6-diamidino-2-phenylindole; CD68, Microsialin; iNOS, Inducible nitric oxide synthase; NOx, Nitrate/nitrite, A.U., arbitrary units.

Apart from its systemic effects, whether cardiac PGC-1α induction is associated with cardiac-specific anti-inflammatory effects in aged T2D hearts is unclear. In our study, F4/80+ macrophage infiltration increased in *db/db* mice hearts compared to the *Wt* counterparts, and such infiltration was attenuated by exercise (Figure 4a and b). This attenuation of cardiac-specific inflammatory cell infiltration was reflected by lower microsialin expression (CD68; macrophage/monocyte marker) and TNFα mRNA expression (Figure 4c) in exercised *db/db* mice hearts. In addition to TNFα, protein levels of cardiac inducible nitric oxide synthase (iNOS) (Figure 4d), which is a common proinflammatory marker, and total cardiac nitrate/nitrite (NOx) levels (Figure 4e) were both increased in sedentary *db/db* hearts and were lowered by short term exercise.

### 3.3 Short-term Exercise-induced PGC-1 Expression Augments Mitochondrial DNA Density but Impairs the Expression of Downstream Transcriptional Activators and Respiratory Enzymes in *db/db* Hearts

**Figure 5:**
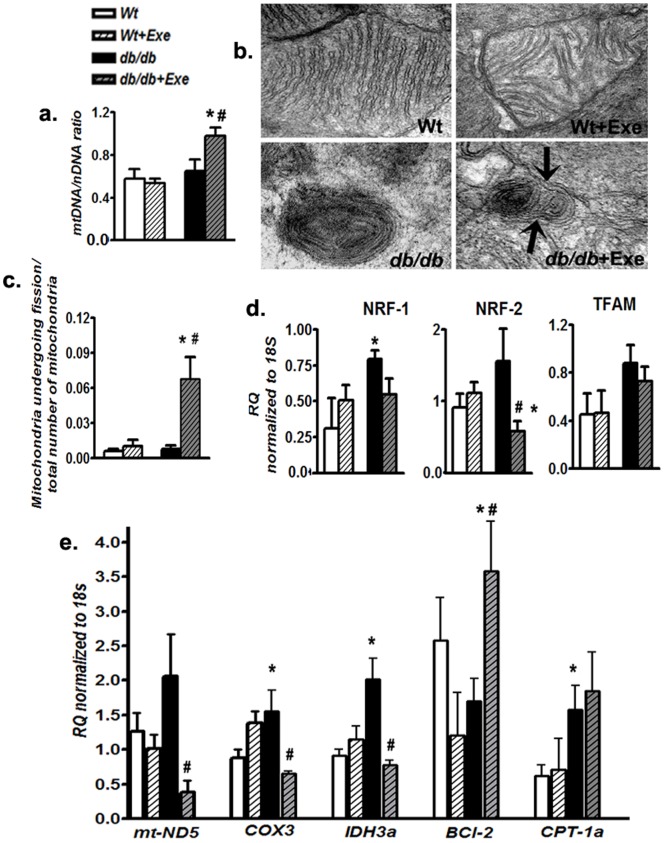
Induction of PGC-1α is associated with an increase in mtDNA density, mitochondrial fission but not downstream transcriptional mediator and respiratory gene expression. (a) Cardiac mitochondrial density was measured as a ratio of mitochondrial DNA (mtDNA) to nuclear DNA (nDNA) using real time PCR. (b) Representative treansmission electron micrographs from myofibrillar sections of the heart demonstrating normal mitochondria in Wt and Wt+Exe groups, damaged mitochondria from sedentary *db/db* hearts and damaged mitochondria undergoing fission/fusion (characterized by dual grooves, black arrows) in *db/db*+Exe mice hearts. Magnification: 16000×. (c) Quantification of total mitochondria undergoing fission expressed per mitochondria from heart sections. (d) mRNA levels of NRF-1, NRF-2 and TFAM in the heart as measured using real time PCR and normalized to 18S RNA. (e) Expression of mitochondrial enzyme transcripts (mt-ND5, COX3, IDH3a) as well as mitochondrial membrane proteins (BCL-2, CPT-1a) was measured using real time PCR and normalized to 18S RNA. Data was analyzed using two-way ANOVA with Bonferroni tests, *p*<0.05 (n = 6). *P<0.05 versus corresponding *Wt* group; ^#^P<0.05 versus corresponding sedentary group. Abbreviations: mtDNA, Mitochondrial DNA; nDNA, Nuclear DNA; NRF1, Nuclear respiratory factor 1; NRF2, Nuclear respiratory factor 2; TFAM, Transcription factor A, mitochondrial; mt-ND5, Mitochondrially encoded NADH dehydrogenase 5; COX3, Cyclooxygenase 3; IDH3a, Isocitrate dehydrogenase 3 (NAD+) a; BCL2, B cell leukemia 2; CPT-1a, Carnitine palmitoyltransferase 1a.

As PGC-1α was upregulated, we next investigated the level of mtDNA as a measure for mitochondrial biogenesis. The level of mtDNA was not different between sedentary Wt and sedentary *db/db* mice hearts (Figure 5a). Short-term moderate intensity exercise had no effect in Wt mice hearts but increased mtDNA levels in *db/db* mice in parallel to an increase in PGC-1α levels (Figure 5a). Next, we performed transmission electron microsopy to evaluate cardiac mitochondrial ultrastructure. Wt and Wt+Exe mice hearts demonstrated normal mitochondria with discrete cristae, whereas both *db/db* and *db/db*+Exe groups demonstrated many abnormal, condensed mitochondria with concentric cristae resembling those observed in patients with mtDNA damage [23] (Figure 5b). Overall, a substantial proportion of total mitochondria in hearts from *db/db*+Exe demonstrated signs of fission/fusion (Figure 5c) that was characterized by dual grooves on the damaged mitochondria and elongated mitochondrial membranes (black arrows, 5 b). As PGC-1α was upregulated, the expression of downstream transcription factors for PGC-1α such as nuclear respiratory factor 1 and 2 (NRF-1 and NRF-2), and transcription factor A, mitochondrial (TFAM) were evaluated [24]. mRNA levels of these downstream transcription factors were either downregulated or remained unchanged in exercised *db/db* hearts (Figure 5d).

In light of a mismatch beween PGC-1α and associated transcription factors, we measured the mRNA levels of three representative mitochondrial genes- NADH dehydrogenase subunit 5 (mt-ND5 from ETC complex I), cytochrome c oxidase subunit 3 (COX3 from ETC complex IV) and isocitrate dehydrogenase (IDH3a from Krebs cycle). There was an uniform attenuation of mRNA levels in the three genes tested (Figure 5e). Despite the downregulation of these three genes, the expression levels of B-cell lymphoma 2 (BCl-2) and carnitine palmitoyl transferase 1 (CPT-1a), which are mitochondrial membrane proteins which are not related to respiratory function, were increased and remained unchanged respectively (Figure 5e). These results suggested that there could be an incomplete transcription of respiratory genes necessary for mitochondrial biogenesis following exercise in *db/db* mice hearts.

### 3.4 Moderate Exercise Augments Mitochondrial Membrane Proteins but not Mitochondrial Oxidative Phosphorylation Complex Subunits in *db/db* Hearts

**Figure 6:**
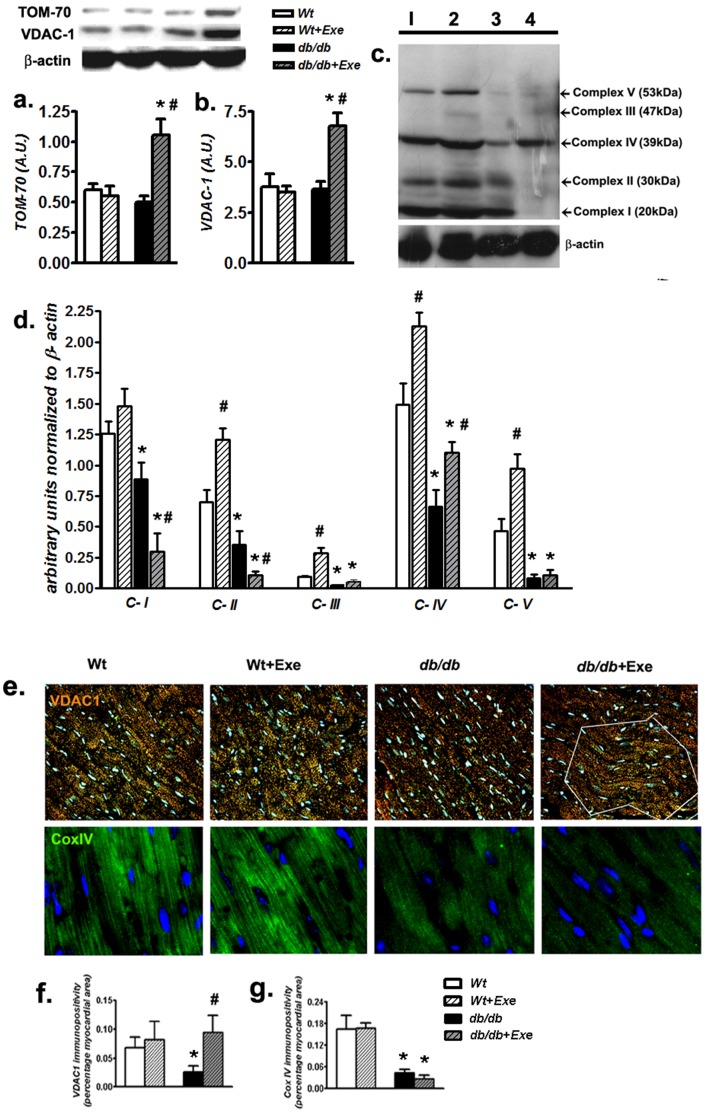
Moderate exercise augments mitochondrial membrane proteins but not mitochondrial oxidative phosphorylation complex subunits in *db/db* hearts. (a) Quantification of cardiac mitochondrial membrane proteins TOM-70 and (b) VDAC-1 by western blotting. Values are expressed as a ratio to β-actin on the same blot. Inset: representative bands of respective proteins from the same blot. (c) Representative lanes from a single western blot demonstrating relative protein levels of complex-1 to 5 in hearts from each group designated as 1 (sedentary Wt), 2 (Wt+Exe), 3 (sedentary *db/db*) and 4 (*db/db*+Exe). Lower band represents β-actin blots from the same gel after stripping and reprobing. (d) Quantification of mitochondrial enzyme complex subunits to β-actin. Relative ratio is expressed in arbitrary units. (e) Heart sections were stained with VDAC-1 and stained with Dylight 594 labeled secondary Ab and co-stained with DAPI to visualize nuclei. Magnification 200×. VDAC-1 staining was attenuated in sedentary *db/db* mice. However, exercised *db/db* mice demonstrated sporadic areas of augmented VDAC-1 staining in specific regions of the ventricular tissue (indicated by white boundary line in *db/db*+Exe. Heart sections were also stained with COX IV, and stained with Dylight 488 labeled secondary Ab. Magnification 600×. COX IV staining was significantly reduced compared to *Wt* in sedentary *db/db* mice, which remained unchanged following exercise. (f) Quantification of VDAC-1 immunopositivity in heart sections as a percentage of total cardiac surface area (g) Quantification of COX IV immunopositivity in heart sections as a percentage of total cardiac surface area. Data was analyzed using two-way ANOVA with Bonferroni tests, *p*<0.05 (n = 6). *P<0.05 versus corresponding *Wt* group; ^#^P<0.05 versus corresponding sedentary group. Abbreviations: TOM-70, mitochondrial import receptor TOM subunit 70 kDa; VDAC, Voltage-dependent anion channel, CI, Complex I; CII, Complex II; CIII, Complex III; CIV, Complex IV; CV, Complex V; COXIV, Cytochrome c oxidase subunit IV.

Based on results in Figure 5, we examined whether the mismatch between respiratory and membrane mRNA levels would be evident at the translational level. Protein expression of mitochondrial specific integral membrane proteins such as the mitochondrial import receptor subunit TOM70 (Figure 6a) and voltage dependent anion channel (VDAC-1) (Figure 6b) were unaffected by diabetes or exercise. However, the expression of these proteins was increased by nearly two-fold in hearts of exercised *db/db* mice (Figure 6a and b). To clarify the impact on mitochondrial respiratory components, we then estimated protein levels of specific electron transport chain subunits from all of the five oxidative phosphorylation complexes by western blotting (Figure 6c and d). As predicted, compared to *Wt* hearts, a specific loss in all of these subunits were apparent in sedentary *db/db* mice (Figure 6c and d). Exercise in Wt hearts increased all of these subunits (Figure 6c and d). Interestingly, the expression patterns of these subunits in *db/db*+Exe mice hearts were varied, with complex I and II demonstrating a decrease and only complex IV showing an increase in expression of the subunit (Figure 6c and d). Expression of cardiac complex III and V remained unchanged and demonstrated a very low level of expression which is similar to its sedentary *db/db* counterparts (Figure 6c and d). To confirm the relative abundance of mitochondrial membrane proteins and respiratory enzymes, we performed immunostaining for a representative mitochondrial outer membrane protein [the voltage dependent anion channel (VDAC-1/porin)], and a representative mitochondrial respiratory subunit [cytochrome oxidase, subunit 4 (COXIV)] on cardiac sections. (Figure 6e, top and lower panels). Compared to *Wt* hearts, a specific loss in VDAC-1 staining was noted in sedentary *db/db* mice that increased following exercise (bordered by white lines in Figure 6e, quantification in 6 f). In contrast, COX IV immunofluorescence was also reduced in sedentary *db/db* hearts but did not change following exercise intervention (Figure 6e and g).

### 3.5 Moderate Intensity Exercise in Aged Diabetic Hearts Induces Oxidative Stress

**Figure 7:**
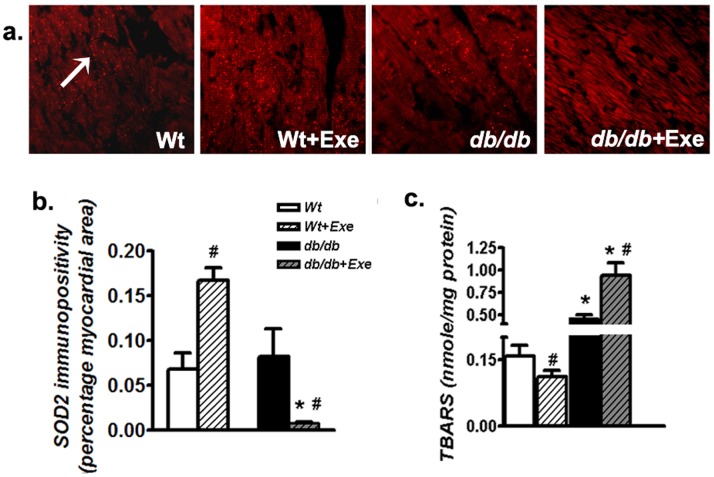
Moderate intensity exercise in aged diabetic hearts induces mitochondrial oxidative stress in *db/db* hearts. (a) Representative micrographs of SOD2 immunofluorescence from heart sections. Please note the red punctate staining for SOD2 for all groups except *db/db*+Exe hearts. Magnification: 200×. (b) Quantification of SOD2 immunopositivity in heart sections as a percentage of total cardiac surface area. (c) TBARS levels in the heart as determined by biochemical assays. Data was analyzed using two-way ANOVA with Bonferroni tests, *p*<0.05 (n = 6). *P<0.05 versus corresponding *Wt* group; ^#^P<0.05 versus corresponding sedentary group. Abbreviations: SOD2, mitochondrial isoform of superoxide dismutase; TBARS, thiobarbituric acid reactive substances.

An immediate cause of improper PGC-1α signaling could be related to ongoing mitochondrial oxidative stress in *db/db* mice hearts following exercise [25]. This conclusion is supported by our previous study which demonstrated augmented oxidative stress in aged *db/db* mice undergoing moderate exercise [16]. In this study, the distribution of superoxide dismutase (SOD2), which functions as a primary mitochondrial antioxidant, was lowered specifically following exercise in *db/db* hearts and were increased in Wt hearts (Figure 7a and b). In parallel, oxidative stress biomarker, thiobarbituric acid reactive substances (TBARS) (Figure 7c), increased in *db/db* mice hearts undergoing exercise in contrast to 8-month old Wt mice where exercise lowered TBARS levels (Figure 7c).

### 3.6 Augmented Oxidative Stress Upregulates PGC-1α but Inhibits Cardiomyocyte Inflammatory Gene Expression in a Dose-dependent Manner

**Figure 8:**
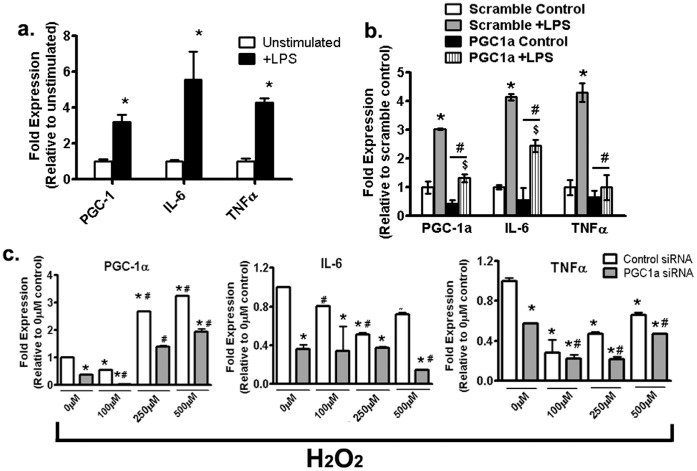
Augmented oxidative stress upregulates PGC-1α and inhibits cardiomyocyte inflammatory gene expression in a dose-dependent fashion. (a) PGC-1α, IL-6 and TNFα gene expressions in H9c2 cardiomyocytes with or without 200 ng/ml LPS. Data was analyzed with two-way Anova with Bonferroni tests, *P<0.05 versus corresponding +LPS (b) PGC-1α, IL-6 and TNFα gene expressions after transfection with either scrambled siRNA or siRNA targeted against PGC-1α. Data was analyzed with two-way ANOVA with Bonferroni tests, *P<0.05 versus scrambled control; ^#^P<0.05 versus scrambled+LPS; ^$^P<0.05 corresponding PGC1α control versus PGC1α+LPS. (c) Effect of increasing doses of H_2_O_2_ on PGC1α, IL-6 and TNFα expressions in the presence or absence of PGC1α knockdown. Experiments were done at least twice in triplicates. Data was analyzed using two-way ANOVA with Bonferroni tests, *P<0.05 versus corresponding 0 µM H_2_O_2_ with scrambled control; ^#^P<0.05 versus 0 µM H_2_O_2_ with PGC1α knockdown. Abbreviations: PGC1α, peroxisome proliferator-activated receptor gamma coactivator 1-alpha; IL-6, interleukin 6; TNFα, tumor necrosis factor alpha; LPS, lipopolysaccharide.

In order to elucidate the relationship between PGC-1α and and its role in cardiac inflammation, we treated differentiated H9c2 cardiomyocytes with 200 ng/ml LPS. This dose has been previously demonstrated to augment inflammation but not cause toxicity in H9c2 cells [26]. LPS treatment upregulated PGC1α, along with IL-6 and TNF-α in cardiomyocytes (Figure 8a). Next, H9c2 cells were treated with siRNA directed towards PGC1α or scrambled siRNA as controls and subjected to LPS treatment (Figure 8b). Partially expressed PGC1α attenuated IL-6 and TNF-α expression (Figure 8b) even at 60% knockdown, demonstrating a crucial role for PGC1α in controlling these pro-inflammatory genes in these cells. To stimulate oxidative stress, H9c2 cells were incubated with H_2_O_2_ which inhibited PGC1α expression at 100 µm H_2_O_2_ concentration but augmented expression with higher H_2_O_2_ doses (Figure 8c). Simultaneously, higher H_2_O_2_ doses did reduce expression of IL-6 and TNF-α (Figure 8c). Knockdown of PGC1α lowered IL-6 expressions across all doses of H_2_O_2_ tested (Figure 8c), whereas TNF-α expressions were reduced at 0, 250 and 500 µM H_2_O_2_ concentrations compared to their scrambled controls (Figure 8c). Irrespective of individual variations at specific doses of H_2_O_2_, gene expressions of IL-6 and TNFα were reduced with elevated oxidative stress compared to 0 mM H_2_O_2_ controls. Cellular viability was assessed by the resazurin assay, which demonstrated no difference in H9c2 viability after 24 hours with either 200 mg/ml LPS or any thes H_2_O_2_ doses (Figure S1 in File S1).

### 3.7 Augmented Oxidative Stress Inhibits Cardiomyocyte Expression of Downstream Transcription Factors and Mitochondrial DNA

**Figure 9:**
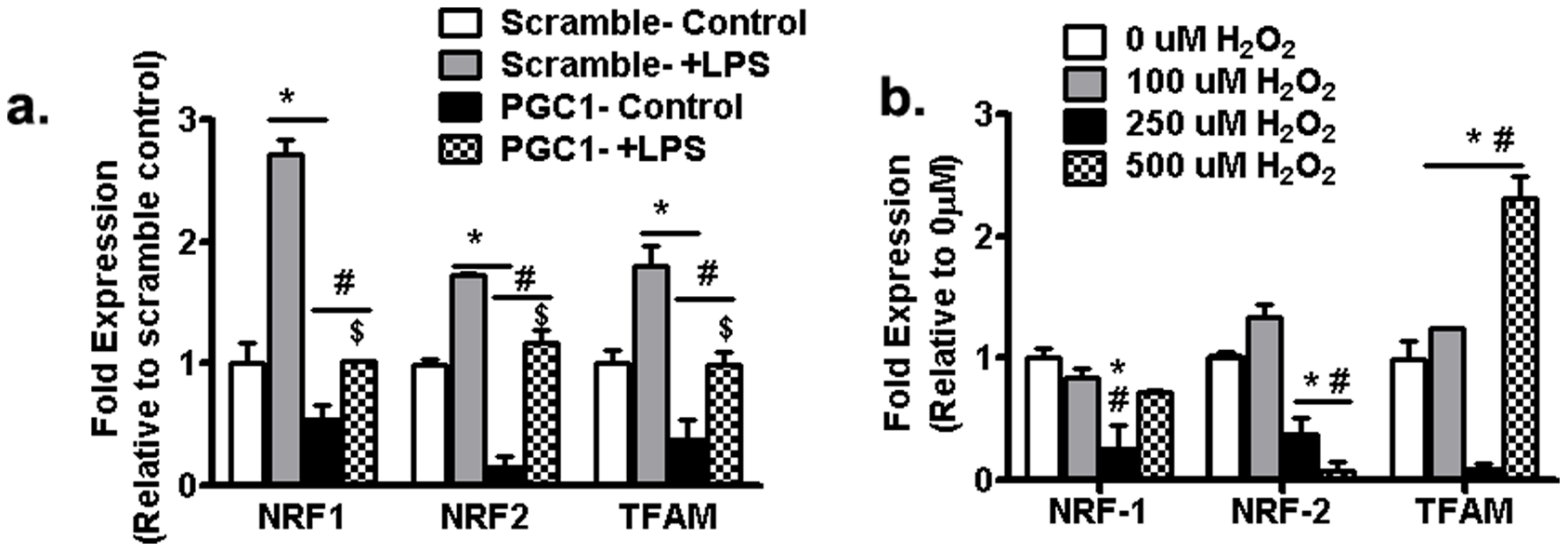
Effect of PGC1α and oxidative stress on downstream transcription factor expression in H9c2 cells. (a) NRF-1, NRF-2 and TFAM gene expressions after transfection of H9c2 cardiomyocytes with either scrambled siRNA or siRNA targeted against PGC-1α. Data was analyzed with two-way ANOVA with Bonferroni tests, *P<0.05 versus scrambled control; ^#^P<0.05 versus scrambled+LPS; ^$^P<0.05 corresponding PGC1a control versus PGC1a+LPS. (b) Effect of increasing doses of H_2_O_2_ on NRF-1, NRF-2 and TFAM gene expressions in H9c2 cardiomyocytes. Experiments were done at least twice in triplicates. Data was analyzed using two-way ANOVA with Bonferroni tests, *P<0.05 versus corresponding 0 µM H_2_O_2_; ^#^P<0.05 versus 100 µM H_2_O_2_. Abbreviations: PGC1a, peroxisome proliferator-activated receptor gamma coactivator 1-alpha; NRF1, Nuclear respiratory factor 1; NRF2, Nuclear respiratory factor 2; TFAM, Transcription factor A; LPS, lipopolysaccharide.

Like inflammatory genes, LPS treatment upregulated NRF-1, NRF-2 and TFAM expression in cardiomyocytes, whereas PGC1α siRNA reduced their expression levels (Figure 9a), indicating that in this cell model, induction of downstream transcription factors are also under the control of PGC1α expression (Figure 9a). When H9c2 cells were incubated H_2_O_2_ to simulate oxidative stress, low levels of H_2_O_2_ (100 µM) did not influence the expression of any of these transcriptional mediators. However, increased doses of H_2_O_2_ (250 and 500 µM) reduced NRF-1 and NRF-1 but increased TFAM expressions (Figure 9b).

## Discussion

The role of PGC-1α in elderly T2D hearts in the absence of improvements in glycemic parameters in either humans or animal models remains unclear. This is because PGC-1 is believed to be integrated with the insulin signaling pathway [14] and elevated insulin levels were found to suppress PGC-1α promoter activity [15]. Most studies demonstrating an increase in PGC-1α activity has been performed in younger animals, with an intense exercise regimen, which lowers insulin levels in T2D by improving insulin sensitivity. Using 8-month old *db/db* mice with diabetes for at least 22 weeks, the goal of this study was to investigate the relationship between induction of PGC-1α on cardiac inflammation and mitochondrial protein expressions in the absence of changes in blood glucose or insulin. A moderate intensity exercise regimen was chosen because this regimen is ineffective in changing glycemic parameters in *db/db* mice, as demonstrated in our earlier studies [12], [27]. It should be pointed out that in those studies a longer exercise regimen for 7–8 weeks were employed, which induced a 10% loss in body weight. The exercise regimen in this study was for a much shorter period of 3 weeks and did not lead to a loss of body weight in aging *db/db* mice.

Without altering body weight, circulating insulin or blood glucose, PGC-1α gene and protein expression increased in the myocardium of aged, exercised diabetic mice. Such an exclusive upregulation of PGC-1α in *db/db* hearts post-exercise does also implicate hyperglycemia as a biogenetic stimulus, as that demonstrated recently in diabetic neurons [28]. Interestingly, PGC-1α expression was not significantly different between sedentary Wt and sedentary *db/db* hearts which is contrary to the earlier findings in 8–9 week old *db/db* mice hearts [29]. This discrepancy could be explained by an advanced age of mice (35–36 weeks) in this study as basal PGC-1α expression levels are known to fluctuate with age [25]. The lack of increase in cardiac PGC-1α response to moderate exercise over 3 weeks in Wt mice was expected, since a higher intensity and duration of exercise (e.g. 7 km running/day) are required for normoglycemic mouse hearts to undergo mitochondrial biogenesis [30]. Chronic periods of exercise (upto 12 weeks) can also fail to induce PGC-1α protein expression in Wt mice [31].

The relationship among PGC-1α, inflammation and mitochondrial biogenesis in the heart with or without diabetes is complex. For example, in normoglycemic hearts, lipopolysaccharide (LPS) induces systemic inflammation, represses PGC-1α signaling [32] but augments the cardiac mitochondrial number [33]. In type 2 diabetic hearts, leptin deficiency increases inflammation [34] but is linked to an elevation of cardiac PGC-1α and mitochondrial biogenesis in mice [29]. In this study, short-term PGC-1α induction in exercised aging diabetic hearts was associated with a robust reduction in systemic inflammation as evident from lower circulating proinflammatory cytokines such as IL-1α, G-CSF, IL-6 and TNFα and a parallel reduction in chemokines such as CCl2, CXCL2 and CXCL10 in the absence of any changes in blood glucose or insulin. These data are in agreement with a previous studies in elderly humans [35], [36] which demonstrated that moderate intensity exercise is sufficient to reduce plasma inflammatory markers in diabetes without changing blood glucose levels. We also demonstrate a decrease in cardiac-specific F4/80+ macrophage infiltration, as well as microsialin and TNFα mRNA expression in exercised *db/db* mice. In diabetes, activated macrophages generate pathological levels of nitric oxide (NO) by inducible nitric oxide synthase (iNOS) [37]. Both iNOS and total cardiac nitrate/nitrite (NOx) levels were also lowered in exercised *db/db* mice hearts.

Mitochondrial biogenesis is often viewed as a compensation for mitochondrial damage during chronic inflammatory states such as diabetes [38]. In this study, electron micrographic evidence of mitochondrial fission/fusion and an elevated mtDNA/nDNA ratio in aging *db/db* mice hearts follwoing exercise was evident. However, such a response resembling biogenesis was not matched with an increase in downstream transcriptional activator expression to PGC-1α such as NRF-1, 2 and TFAM. In tandem with impairment of these downstream transcriptional activators, mt-ND5, COX3 and IDH3a, genes related to respiratory functions were downregulated in exercised, aged *db/.db* mice hearts. Despite such downregulation of respiratory genes, expression of mitochondrial membrane proteins which are *not* related to respiratory functions, such as BCl-2 or CPT-1a, either increased or remained unchanged, which indicated differential influence on specific groups of genes. Beyond the gene level, this curious relationship was also verified at the protein level by western blotting and immunofluorescence demonstrating an increase in mitochondrial membrane/structural proteins like VDAC-1 and TOM70 but a loss of oxidative phosphorylation subunit enzymes in aged *db/db* hearts undergoing exercise. In this regard, human T2D has previously been associated with an elevation in muscle VDAC-1 in the absence of mitochondrial respiratory complexes [39].

An immediate cause of derangement of the mitochondrial biogenetic program driven by PGC-1α could be related to an increased oxidative stress. Deficiency in respiratory subunits as that seen in *db/db* mice hearts undergoing exercise, could provoke increased leakage of electrons, initiating increased free radical generation from the mitochondria [40]. This is supported by our earlier study which demonstrated augmented oxidative stress in aged *db/db* mice undergoing short-term, moderate intensity exercise [16]. In this study, distribution of SOD2, a vital mitochondrial antioxidant was reduced and TBARS increased in sedentary *db/db* mice, which worsened with exercise. In contrast, Wt hearts demonstrated a rapid increase in cardiac SOD2 and a reduction in TBARS following the same exercise regime. This is in agreement with previous studies demonstrating a beneficial effect of moderate exercise on cardiac antioxidant levels in normoglycemic mice [41].

Although surprising, an increase in mitochondrial membrane protein levels without a corresponding increase in mitochondrial respiratory enzymes is not unprecedented. Moderate exercise regimen has been previously associated with dissociation of PGC-1α and respiratory subunit expression leading to ‘empty’ cristae within the mitochondria, in lieu of functional mitochondrial respiratory subunits in skeletal muscle [42]. Such abnormalities have also been reported with an advanced age, characterized by elevated oxidative stress, where PGC-1α may not be able to improve mitochondrial biogenesis following exercise intervention [43]. An earlier onset and a long duration of diabetes (as that seen in *db/db* mice) may be another important factor in determining mitochondrial plasticity [39].

In order to establish an inter-relationship between a possible role for oxidative stress, PGC-1α, proinflammatory cytokines and downstream transcription factor signaling, we utilized H9c2, a rat clonal cell line which has been used to study mitochondrial biogenesis and inflammation [44], [45]. Using LPS as an inflammatory stimulus, which has been associated with obesity, inslin resistance and T2D [46] and siRNA against PGC-1α, we demonstrate that LPS increases PGC-1α mediated expression of pro-inflammatory cytokines like IL-6 and TNFα and downstream mediators like NRF-1, NRF-2 and TFAM in H9c2 cardiomyocytes.

However, with our *in vivo* data, we had demonstrated an inverse association of PGC-1α with inflammatory cytokines and genes in exercised *db/db* hearts in this study. Therefore, additional confounders must exist to explain this discrepancy. In this regard, we speculated a potential role of oxidative stress which is increased in this model and is known to be upregulated following exercise, aging or chronic diabetes [16]. Utilizing various doses of H_2_O_2_ between 100–500 µM on H9c2 cells with or without PGC-1α knockdown, we demonstrate that PGC-1α expression is inhibited at a low (100 µM) but increased at higher concentrations of H_2_O_2_ as demonstrated previously [47]. Interestingly, with regard to both cytokines and downstream transcriptional mediators, high PGC-1α expression levels at elevated concentrations of H_2_O_2_ led to an overall downregulation of expression, except for TFAM. Expression of TFAM increased by almost 2.5 folds under the highest dose of H_2_O_2_. In previous studies, such induction of TFAM specifically at elevated oxidative stress has been noted to maintain mitochondrial DNA integrity [48]. In agreement with our *in vitro* data, both sedentary and exercised *db/db* hearts demonstrated no decreases in either TFAM expression or mtDNA levels even in the face of elevated oxidative stress compared to Wt hearts. We believe that low levels of oxidative stress (as demonstrated in Wt mice hearts or 100 µM H_2_0_2_ in H9c2 cells) may be responsible for a reduced PGC-1α expression, which could still be sufficient to induce downstream transcription mediators and initiate mitochondrial respiratory proteins synthesis as demonstrated in Wt hearts However, elevated oxidative stress impairs downstream gene transcription activity of PGC-1α which could in turn upregulate PGC-1α expression at higher H_2_O_2_ concentrations as a compensatory mechanism.

In summary, we demonstrate that short-term augmentation of PGC-1α in the absence of alterations in glycemic parameters can attenuate both systemic and cardiac inflammation. Our *in vitro* data supports the idea that elevations in oxidative stress could play a significant role in attenating proinflammatory gene expressions following exercise in the aging *db/db* mice hearts. However, such an exercise regimen by the same mechanism can also provoke a decoupling of upstream cardiac PGC-1α and downstream transcription factors like NRF-1 and NRF-2, leading to an impaired mitochondrial respiratory protein expression. These data could also imply that either a longer duration of PGC-1α induction [49] or an improvement in glycemic parameters (as that observed in other insulin-sensitive tissues [6], [7]) or anti-oxidant supplementation are necessary before complete mitochondrial benefit is achieved in aging diabetic hearts. It is also possible that moderate intesity exercise, although sucessful in initiating antioxidant responses in either normoglycemic mice [41] or in younger *db/db* hearts [50], is incapable of provoking a successful biogenetic response in aged diabetic hearts and a more intense training regimen could be needed [6]. This is supported by a superior effect of high intensity exercise compared to moderate exercise in amelioration of cardiovascular pathologies in humans [51].

## Supporting Information

File S1 **Supplementary tables and figure.**
(DOC)
