# Supplementary material for: Short Term Exercise Induces PGC-1α, Ameliorates Inflammation and Increases Mitochondrial Membrane Proteins but Fails to Increase Respiratory Enzymes in Aging Diabetic Hearts
Source: PLoS One. 2013 Aug 1;8(8):e70248. doi: 10.1371/journal.pone.0070248 (PMC3731348; doi:10.1371/journal.pone.0070248)

**Table S1.** **Primer sequences used for quantification of mouse cardiac mRNA levels by qPCR.** Both the forward primer (F) and reverse (R) primers are indicated.

| GENE | PRIMER SEQUENCES  (5’-3’) | GENE BANK  REFERENCE # |
| --- | --- | --- |
| Cluster of Differentiation  68 (CD 68) | F: AGCTGCCTGACAAGGGACACT  R: AGGAGGACCAGGCCAATGAT | NM_009853 |
| Tumor Necrosis Factor  Alpha (TNFA) | F:CATCTTCTCAAAATTCGAGTGACAA  R: TGGGAGTAGACAAGGTACAACCC | NM_013693 |
| Peroxisome proliferator  activated receptor gamma co-activator 1 alpha (PGC1a) | F: TTGCCCAGATCTTCCTGAAC  R: TCTGTGAGAACCGCTAGCAA | NM_008904 |
| Nuclear respiratory factor 1  (NRF 1) | F: TGAGGTCGAATGGTATGTGG  R: AGGACTGAAAGCAGCGTCTC | NM_010938 |
| Nuclear respiratory factor 2  (NRF 2) | F: CTCAGCATGATGGACTTGGA  R: CCTGTTCCTTCTGGAGTTGC | NM_010902 |
| Transcription factor A, mitochondrial (TFAM) | F: AGTTCATACCTTCGATTTTC  R: TGACTTGGAGTTAGCTGC | NM_009360 |
| Mitochondrially encoded NADH dehydrogenase 5  (mt-ND5) | F: ACAGCTATTTGTGCCCTCACCCAA  R: GCATGGCTTTGAAGAATGCGTGGG | XM_003086366 |
| Cytochrome c oxidase  subunit 3 (COX-3) | F: CCTACCAAGGCCACCACACTCCT  R: TTCCTGTTGGAGGTCAGCAGCC | AK131579 |
| Isocitrate Dehydrogenase 3a (IDH-3a) | F:CGC GTG GGT GTC CAA GGT CTC  R:TGT GAC ATT GCG CTC CTC CAA | NM_029573 |
| Beta Cell Lymphoma 2  (BCL2) | F: CTGGTGGACAACATCGCTCTG  R: GGTCTGCTGACCTCACTTGTG | NM_009743 |
| Carnitine palmitoyltransferase 1a (CPT1a) | F:ACTCCTGGAAGAAGAAGTTCA  R:AGTATCTTTGACAGCTGGGAC | NM_013495 |
| 18s ribosomal RNA  (18S r RNA) | F: CGGCTACCACATCCAAGGAA  R: GCTGGAATTACCGCGGCT | NR_003278 |

**Table S2.** **Primer sequences used for quantification of H9c2 mRNA levels by qPCR.** Both the forward primer (F) and reverse (R) primers are indicated.

| GENE | PRIMER SEQUENCES  (5’-3’) | GENE BANK  REFERENCE # |
| --- | --- | --- |
| Peroxisome proliferator  activated receptor gamma co-activator 1 alpha (PGC1a) | F: ACATGCTCAAGCCAAACC  R: CAGTTCCAGAGAGTTCCACAC | NM_031347.1 |
| Nuclear respiratory factor 1  (NRF 1) | F: CCTCCGTCTCCTTCTTTCTAGC  R: TTTGGGTCACTCCGTGTTCC | NM_001100708.1 |
| Nuclear respiratory factor 2 (NRF 2) | F: ATGACCATGAGGTTTCTTCAC  R: CTCCATGTCCTGCTGTATG | NM_031789.2 |
| Transcription factor A, mitochondrial (TFAM) | F: AGCTAAACACCCAGATGC  R: GCTGCTCTTTATACTTGCTCAC | NM_031326.1 |
| Interleukin 6 (IL-6) | F: TTCTCTCCGCAAGAGACTTC  R: GGTCTGTTGTGGGTGGTATC | NM_012589.2 |
| Tumor Necrosis Factor  Alpha (TNFA) | F: CAACAAGGAGGAGAAGTTCC  R: GATCTGAGTGTGAGGGTCTG | NM_012675.3 |
| 18s ribosomal RNA  (18S r RNA) | F: TTGATTAAGTCCCTGCCCTTTGT  R: CGATCCGAGGGCCTAACTA | NR_046237.1 |

**Supplementary Methodology**

**Resazurin flurometric assay for H9c2 viability**. Resazurin (from Biotium, also called Alamar Blue™) is a dye that is non-fluorescent but is converted into fluorescent resofurin once it is reduced in viable mitochondria of living cells. Briefly, 1:10 dilution of resazurin solution was added to H9c2 cells in each well of a 96-well plate. The plate was then incubated at 37 degrees with 5% CO2 for 2 hours. Fluorescence was measured by using a Promega Glomax plate reader in the fluorescent mode with an excitation of 525nm and emission of 580-640 nm. Fluorescence units were normalized to control cells with no H_2_O_2_ or LPS and expressed in arbitrary units.

**Figure S1. Cell viability data using Resazurin as described above. There was no statistical difference between control H9c2 cells with or without LPS and H_2_O_2_ at the indicated doses**


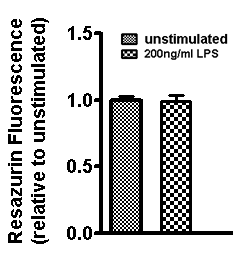

Supplement: File S1 — Supplementary tables and figure. (DOC) [file pone.0070248.s001.doc]
